# Fluid mechanical performance of ureteral stents: The role of side hole and lumen size

**DOI:** 10.1002/btm2.10407

**Published:** 2022-09-13

**Authors:** Shaokai Zheng, Dominik Obrist, Fiona Burkhard, Francesco Clavica

**Affiliations:** ^1^ ARTORG Center for Biomedical Engineering Research, Faculty of Medicine University of Bern Bern Switzerland; ^2^ Department of Urology Inselspital, Bern University Hospital, University of Bern Bern Switzerland

**Keywords:** calibration, encrustation, micro‐PIV, modeling, particle image velocimetry, shear stress, ureter, ureteral stent

## Abstract

Ureteral stents are indispensable devices in urological practice to maintain and reinstate the drainage of urine in the upper urinary tract. Most ureteral stents feature openings in the stent wall, referred to as side holes (SHs), which are designed to facilitate urine flux in and out of the stent lumen. However, systematic discussions on the role of SH and stent lumen size in regulating flux and shear stress levels are still lacking. In this study, we leveraged both experimental and numerical methods, using microscopic‐Particle Image Velocimetry and Computational Fluid Dynamic models, respectively, to explore the influence of varying SH and lumen diameters. Our results showed that by reducing the SH diameter from 1.1 to 0.4mm the median wall shear stress levels of the SHs near the ureteropelvic junction and ureterovesical junction increased by over 150%, even though the flux magnitudes through these SH decreased by about 40%. All other SHs were associated with low flux and low shear stress levels. Reducing the stent lumen diameter significantly impeded the luminal flow and the flux through SHs. By means of zero‐dimensional models and scaling relations, we summarized previous findings on the subject and argued that the design of stent inlet/outlet is key in regulating the flow characteristics described above. Finally, we offered some clinically relevant input in terms of choosing the right stent for the right patient.

## 1 INTRODUCTION

Ureters are long collapsible tubes that transport urine from the kidneys to the bladder. The efficacy of transport, however, can be impeded by a range of congenital and acquired pathological obstructions.^1^ To restore the flow, ureteral stents are inserted to relax and widen the ureter.

The ureteral stents are thin polymer tubes typically 20‐30 cm in length, with circular cross sections of outer diameters ranging between 1.6mm (4.8Fr) and 2.67mm (8Fr). Modern ureteral stents also feature a curl at each end of the stent, known as pigtails, to prevent dislocation of the stent after placement. Annually, there are over 1.5 million stents used worldwide; yet, more than 80% of the patients suffer from stent‐related complications, posing significant threats to the quality‐of‐life of patients and creating economic burdens for the health care system.^2^, ^3^

Among all complications of indwelling stents, the frequent development of biofilm and encrustation remains a key limiting factor of stent efficacy. They are aggregates of conditioning films, bacterial colonies and crystals that start to accumulate on the stent surfaces once in contact with urine. Depending on the actual physicochemical and microbiological environment, the composition may vary, but over time the growing volume will cause secondary stent obstructions that compromise the drainage capacity, and the bacterial content will significantly increase the chance of urinary tract infections.^4^, ^5^ Although many efforts have been made to fight against biofilm and encrustation by means of material design and functional surface coatings, it remains one of the primary challenges in stent development.^2^ Previous clinical observations have drawn attention to the side holes (SHs), which are small openings in the stent wall to facilitate exchange of fluids between the luminal (in the stent lumen) and extraluminal (between stent and ureter walls) spaces, that often end up heavily encrusted or completely occluded.^6^, ^7^, ^8^ Once a SH becomes occluded, flow cavities in the vicinity also aggravate bacterial attachment,^9^ further increasing the risk of infection.

The causal relation between SH and encrustation was investigated recently^10^ using microfluidic chips, and the low shear stress level near the SH was considered the main reason for the accumulation of micro‐particles. The authors^10^ proposed a “streamlined” SH architecture with optimized wall thickness (0.3mm) and vertex angle (45°) that reduced the encrustation rate by approximately 90%. Prior to that, several numerical models exists that examined urine flows in full‐scale ureter models under the impact of different stent geometries and ureter shapes, with and without local obstruction.^11^, ^12^, ^13^ One of the common conclusions was that the fluxes through SHs were strongest near the ureteropelvic junction (UPJ) and the ureterovesical junction (UVJ). Other SHs underwent fluxes only when a local obstruction was present in the vicinity.^14^ A recent study^15^ linked these observations to the inhomogeneous shear stress patterns along the stent and further showed that the shear stress level was lower in the proximal region (close to the UPJ), where more encrustations have been reportedly observed in clinical studies. It seems that the interplay between the large‐scale flow characteristics (e.g., flow rates through SHs) and the small‐scale quantities (e.g., flow patterns near the SH and associated shear stresses) is key to understand the dynamics of the encrustation process.

In this study, we present the first experimental setup of a stented ureter model in full scale, which allows microscopic particle image velocimetry (μ‐PIV) measurements near the SHs at various streamwise locations. Numerical counterparts of the experiments are exploited to investigate further variations of the SH and lumen diameter of the stent. As such, we hypothesize that the flow behaviors in stented ureters in vivo can be explored by means of in vitro studies using simplified models, given that the baseline fluid mechanical principles stay the same, and that the flow characteristics can be manipulated in favor of the stent efficacy by varying the SH and lumen diameter. In Section 3, fluxes through the SHs and the associated shear stresses are presented, and the link between these quantities is discussed. In the discussion, we use simple zero‐dimensional (0D0D) models and scaling relations to summarize the previous conclusions on large‐scale flow characteristics and briefly evaluate the impact of varying parameters. The objective of the current study is to offer a glimpse into the underlining principle of fluid mechanics in the stented ureteric system by means of experimental, numerical, and theoretical results, trying to offer some clinically relevant input in terms of choosing the right stent for the right patient and a practical reference for future stent development.

## 2 MATERIALS AND METHODS

### 2.1 Experiments

#### 2.1.1 Ureter model

**FIGURE 1:**
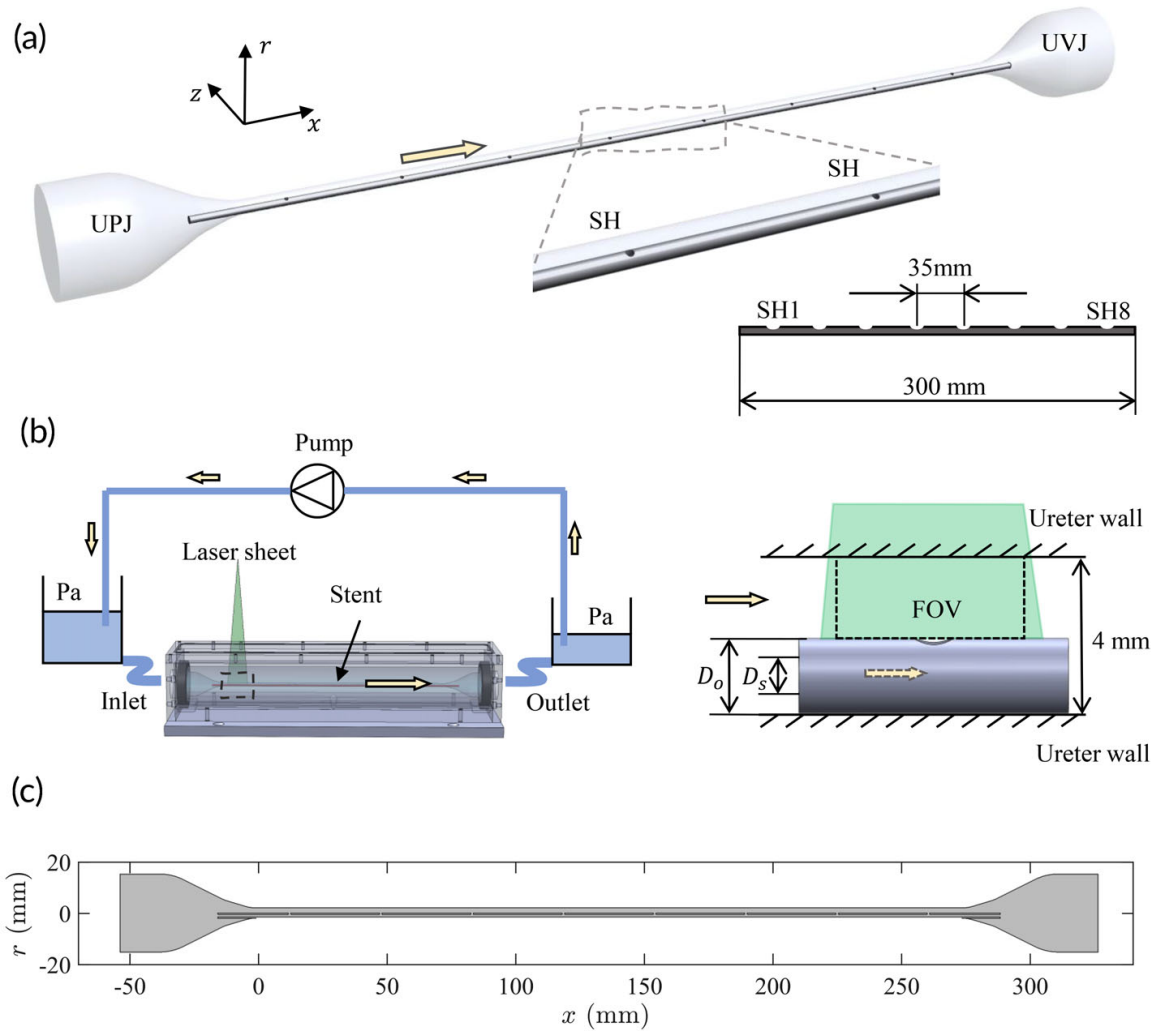
(a) Three‐dimensional model of the ureter used in this study, showing ureteropelvic junction (UPJ) and ureterovesical junction (UVJ). The side holes (SHs) are numbered along the direction from UPJ to UVJ. (b) Schematic sketch of the experimental setup. The field of view (FOV) of the measurement is directly above the stent SH and is aligned with the x−r plane. (c) Two‐dimensional computational domain on the median (x−r) plane of the ureter model.

Previous studies in humans^16^ and in porcine^17^ models have shown that the peristaltic movement of the ureteral wall is reduced or completely stopped after stent placement. The ureter model (the straight part between UPJ and UVJ) in the study was therefore simplified as a rigid tube of L=270.6mm in length and D=4mm in diameter. These dimensions were taken within ranges of previously published clinical data measured from human subjects.^18^ Two extra conical chambers were added at each end of the ureter model to simulate the UPJ and the UVJ, respectively (Figure 1a). The entire model was made in one piece out of Sylgard® 184 silicone elastomer (Dow Corning Corp. MI, USA) by molding. The mixing ratio of silicone to curing agent was 10:1 by weight, and the model was cured at room temperature for 48 h after pouring. These values were chosen as a result of the refractive index matching procedure required to perform the PIV measurements.

For the stent model, commercial stents made of polyurethane were used initially. Stent position, however, was not reproducible due to the flexibility of the stent material. Furthermore, previous numerical studies have shown that the activation of SHs happens mainly in the vicinity of the UPJ and UVI.^11^, ^19^ Therefore, we reduced our stent models to simple straight stainless steel tubes with an outer diameter of $D_{o}=2\;\mathrm{mm}$ (6 Fr) and lumen diameter $D_{s}=1\;\mathrm{mm}$. Two stent models were realized in the experimental study, featuring SHs with diameters of $D_{\mathrm{SH}}=1\;\mathrm{mm}$ and 0.7mm, respectively, connecting the luminal and extraluminal spaces. These SHs were 35mm apart and evenly spaced along the longitudinal axis of the stent (Figure 1a). All SHs were facing up. The origin was defined at the center of the ureter cross section (r=0mm), and at the beginning of the ureter model (x=0mm, Figure 1c). The stent model extended beyond the ureter model by 14.7mm on each end. As such, the first SH (SH1) was situated at x=12.8mm from the inlet of the ureter model and the last one (SH8) at x=257.8mm.

#### 2.1.2 Flow setup

The silicone model was placed in an acrylic box, where the two ends (i.e., inlet and outlet) were connected through Luer tapers to external reservoirs via silicone tubes (Figure 1b). A steady flow was established by the pressure difference in the two reservoirs and was regulated by a peristaltic pump (MCP‐ISM 404; Cole‐Parmer GmbH, GE). To cater the optical measurement, the refractive index of the working fluid was matched to the silicone elastomer using mixtures of glycerol and water with 60% (w/w%) glycerol. The density of the fluid was $\rho^{\exp}=1150\;\mathrm{kg}/m^{3}$ and the kinematic viscosity was $\nu^{\exp}=9.98\times 10^{-6}\;m^{2}/s$, measured by a Ubbelohde viscometer (Type 501‐10; SI Analytics GmbH, Mainz, DE, Germany) at 20°C.

In the human ureter, the physiological Reynolds number can be estimated as $Re^{*}=U_{c}^{*}D^{*}/\nu^{*}\approx 10.55$, where the ureter diameter $D^{*}=4\;\mathrm{mm}$ and viscosity $\nu^{*}=1.005\times 10^{-6}\;m^{2}/s$, and the centerline velocity $U_{c}^{*}=2.65\;\mathrm{mm}/s$ was calculated assuming laminar pipe flow with rigid walls (Poiseuille flow) and a flow rate of $Q^{*}=1\;\mathrm{ml}/\min$. All values were taken from physiological values of the human urinary system.^18^ To match the Reynolds number, the flow rate of the experiment was chosen to be $Q^{\exp}=10.03\;\mathrm{ml}/\min$ as the closest approximation permitted by the pump.

#### 2.1.3 PIV setup

To measure the velocity field, a μ‐PIV system was built. The system consisted of a Nd:YAG continuous laser with 5 W maximum energy (RayPower 5000; DANTEC, Dantec Dynamics, DK) operating at wavelength λ=532nm, a high‐speed CMOS camera (FASTCAM Mini AX100, Photron Europe Ltd, UK) with 1 M pixel equipped with a long‐distance microscope (Infinity K2 with the CF‐2 objective, Infinity Photo‐Optical Co., USA), and optics to produce a laser sheet to illuminate the field of view (FOV) (Figure 1b). A long‐pass filter with passband of 570 nm was mounted in front of the microscope to filter the acquired light. Nearly neutrally buoyant ($\rho_{p}=1180\;\mathrm{kg}/m^{3}$) fluorescent polymethyl methacrylate (PMMA) particles of 20μm diameter (Lab261, CA, USA) were used as tracer particles that excite at λ=530nm and emit at λ=582nm. The response time of the particle was estimated as $\tau_{p}=d_{p}^{2}\rho_{p}/\left(18\mu \right)=2.28\times 10^{-6}\;s$, where $d_{p}$ and $\rho_{p}$ denote the diameter and density of the tracing particle, respectively, and μ is the fluid dynamic viscosity. By taking the centerline velocity of the Poiseuille flow in our ureter model $U_{c}=26.6\;\mathrm{mm}/s$ as characteristic velocity and D=4mm as characteristic length, the Stokes number of the particles was estimated as $\mathrm{Stk}=\tau_{p}U_{c}/D^{*}=1.52\times 10^{-5}$, which was much smaller than 0.1, suggesting a good tracing fidelity.^20^

The acrylic box shown in Figure 1b was mounted on two dovetail optical rails attached to an optical table. The box could therefore traverse along the x direction, covering all SH locations. Based on preliminary studies, interluminal transport of fluid happens mainly near the UPJ and UVJ. Therefore, only SH1, 2, 7, and 8 were included in our final data set.

After calibrating the imaging system using an ex situ procedure (see Supporting Information S1 for further details with code), the RMS error of the re‐projected image was ~0.2px on average (range 0.1‐0.3 px), which incorporated both the calibration error and the residual error caused by the refractive index matching.

During acquisition, the camera was operated at 500Hz, and 20,000 images were acquired at each SH location. After removing the background, an image‐overlapping algorithm was used to enhance the particle image density (see Supporting Information S1 for more details). Velocity fields were calculated with the open source code PIVLab^21^ using the ensemble‐correlation with interrogation window of 64×64px for the first pass and 32×32px for the second, both with 50% overlap. The velocity field was filtered by the outlier detection algorithm from the literature^22^ and filled by the moving median of the 3×3 neighboring points.

### 2.2 Numerical simulations

Numerical counterparts of the same experiments were performed to cross validate and to investigate further stent geometries. In a preliminary study, a full three‐dimensional (3D) numerical simulation was performed and the results showed that velocities in the z direction were negligible on the median plane. As a result, two‐dimensional (2D) models on the median plane of the ureter model were constructed (Figure 1c). The geometry was discretized using second‐order (P2) elements for the velocity components and linear (P1) elements for the pressure field.

The steady‐state incompressible Navier–Stokes equations were solved using the computational fluid dynamics (CFD) module from COMSOL Multiphysics® (v5.6, COMSOL Inc., Stockholm, SE). The density of the fluid was $\rho^{CFD}=1000\;\mathrm{kg}/m^{3}$ with kinematic viscosity of $\nu^{CFD}=1.005\times 10^{-6}\;m^{2}/s$. The no‐slip boundary condition was imposed on all walls. A fully developed flow with mean velocity of 0.235mm/s was given as inlet condition, which produced a centerline velocity of $U_{c}=2.64\;\mathrm{mm}/s$ in the ureter. A static pressure of 0Pa was prescribed at the outlet. The multifrontal massively parallel sparse direct solver (MUMPS) from COMSOL was used to solve the system with a relative tolerance of $10^{-8}$. After a convergence study on the imbalance, flow rate through the first SH, and shear stresses on the stent external wall at x=0mm, a mesh with 1 M elements was generated, giving an uncertainty level smaller than 0.2%.

To evaluate the impact of SH diameter on the activation of SHs, seven cases with $D_{\mathrm{SH}}=0.4,0.5,0.7,0.8,0.9,1$, and 1.1mm were performed, while keeping $D_{S}=1\;\mathrm{mm}$ and all other parameters constant. Based on geometrical considerations, larger SH diameters ($D_{\mathrm{SH}}\gg D_{S}$) were not realistic as the through hole would cut into the stent wall, partially reducing the stent wall thickness and compromising the tensile strength of the stent. The stent lumen size was also investigated by reducing the lumen diameter $D_{S}$ from 1mm to 0.8mm, with four cases of $D_{\mathrm{SH}}=0.4,0.5,0.7$, and 1mm. The outer diameter of the stent $D_{o}$ was kept constant at 2mm.

## 3 RESULTS

### 3.1 Base flow

**FIGURE 2:**
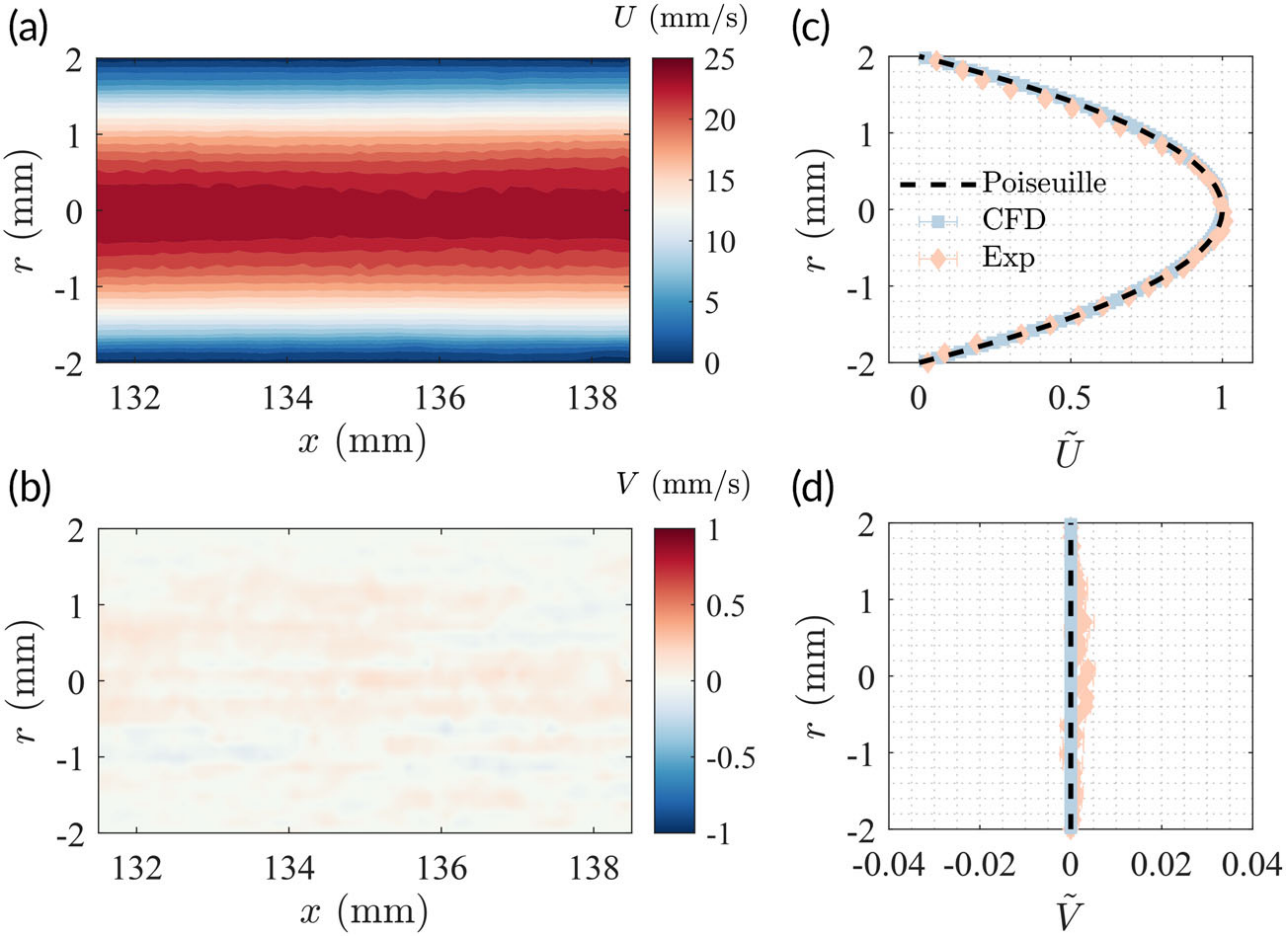
Streamwise (a) and transverse (b) velocities measured experimentally at x=135.3mm on the median plane of the ureter model. The normalized streamwise ($\tilde{U}$) and transverse ($\tilde{V}$) velocities are compared against the computational fluid dynamics (CFD) results and theoretical Poiseuille profile in (c) and (d). The mean and standard deviation are given by the symbol and the error bar, respectively.

To validate both setup, we examine the flow in the ureter model without the inserted stent, which resembles a Poiseuille flow. The mean streamwise (U) and transverse (V) velocity contours experimentally measured around x=135mm are given in Figure 2a,b, respectively. After averaging along the x direction, the velocity profiles from both experimental and numerical results are compared against the theoretical Poiseuille profile, as shown in Figure 2c,d, for the streamwise and transverse velocities, respectively. Estimations of error are calculated as

(1) $$\varepsilon_{u}=\frac{1}{N}\sum_{i=1}^{N}\sqrt{{\left(\tilde{U}\left(r_{i}\right)-{\tilde{U}}^{*}\left(r_{i}\right)\right)}^{2}},\varepsilon_{v}=\frac{1}{N}\sum_{i=1}^{N}\sqrt{{\tilde{V}}^{2}\left(r_{i}\right)}\;$$

where $\tilde{U}=U/U_{c}$, and $U^{*}$ is the theoretical Poiseuille profile. For experimental results, we have $\epsilon_{U}^{\exp}=2.33\%$, and $\epsilon_{V}^{\exp}=0.13\%$, whereas for CFD results the errors are $\epsilon_{U}^{\mathrm{CFD}}=0.08\%$ and $\epsilon_{V}^{\mathrm{CFD}}<0.01\%$. These results serve as a summary of the experimental and numerical uncertainties of the current study.

### 3.2 SH activation

**FIGURE 3:**
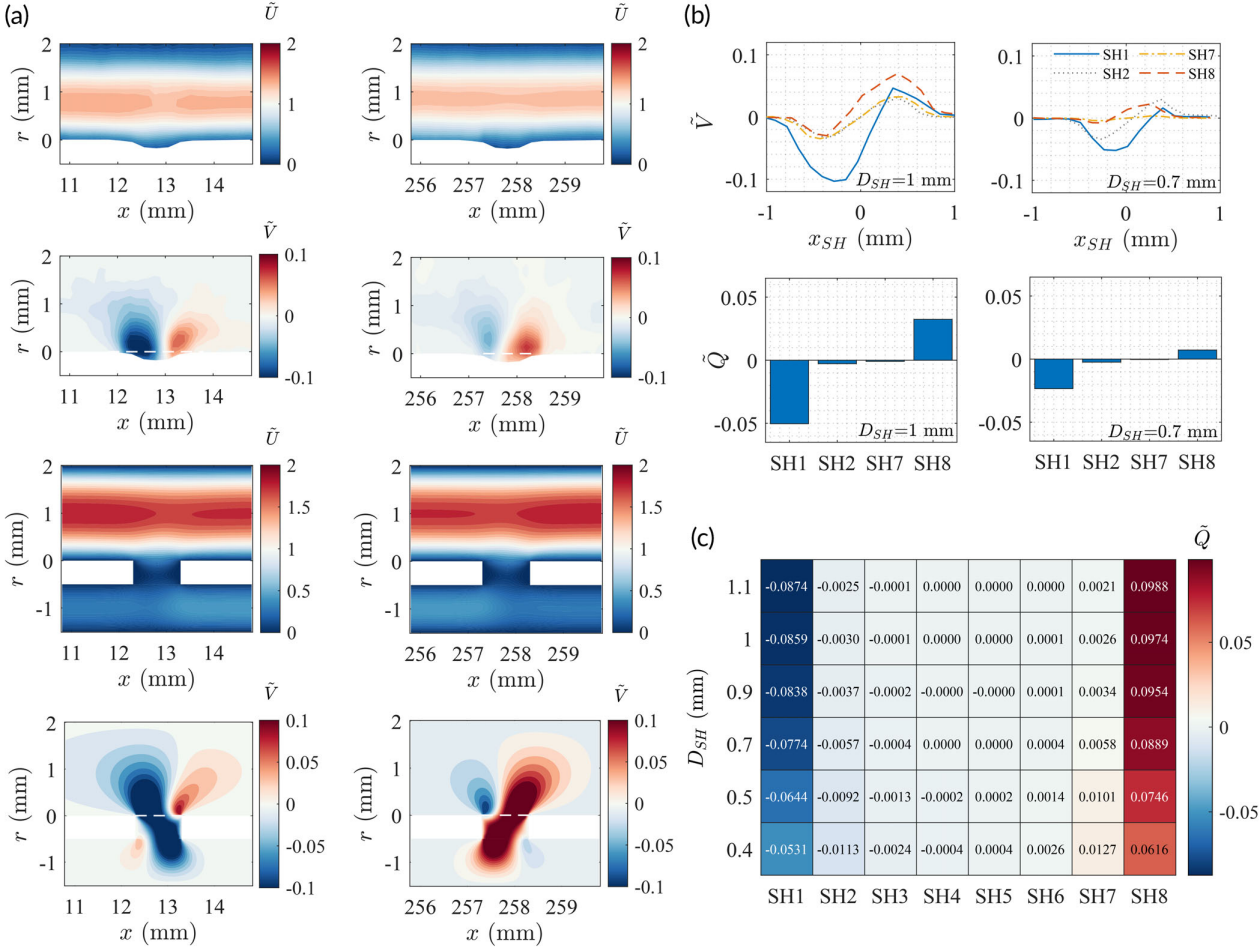
(a) Experimental (upper two rows) and computational fluid dynamics (CFD) (lower two rows) results of the normalized streamwise ($\tilde{U}$) and transverse ($\tilde{V}$) velocities above SH1 (left column) and SH8 (right column) with $D_{\mathrm{SH}}=1\;\mathrm{mm}$. CFD results also show the velocity fields in the lumen of the stent $r\in \left[-1.5,\;-0.5\right]$. The white dashed line shows the location where transverse velocities are extracted for calculations in (b) and (c). (b) Experimental results of the normalized transverse velocity ($\tilde{V}$) profiles and transverse fluxes $\tilde{Q}$ through the SHs, measured along the white dashed lines in (a). (c) CFD results of the transverse fluxes $\tilde{Q}$ at different SHs. Each row represents a given SH diameter $D_{\mathrm{SH}}$.

An activated SH is defined where appreciable interluminal exchange of fluid is present. To visualize this phenomenon, the normalized velocity fields ($\tilde{U}$ and $\tilde{V}$) directly above SH1 and SH8 of the stent with $D_{\mathrm{SH}}=1\;\mathrm{mm}$ are shown in Figure 3a for both experimental and CFD results. It clearly shows the transverse fluxes through the SHs from the extraluminal space into the luminal space at SH1 and vice versa at SH8, as evidenced by the contours of negative and positive transverse velocities, respectively.

To quantify the fluxes, the transverse velocity profiles at r=0mm are extracted from the velocity fields (Figure 3a), and the fluxes through the SHs were calculated as $\tilde{Q}=\int_{-1}^{1}\tilde{V}\left(x_{\mathrm{SH}}\right)dx_{\mathrm{SH}}$, where $x_{\mathrm{SH}}=0$ is at the center of the SH. The experimental results (Figure 3b) show that only SH1 and SH8 are significantly activated. The fluxes through SH2 and SH7 in both stents are much smaller compared to those of SH1 and SH8. The velocities also fall closely to the uncertainty range of the measurement. The CFD results (Figure 3c) are therefore presented to extend the investigation. It is clear that SHs at either end of the stent (SH1 and SH8) are always active, and the magnitude of flux reduces with decreasing SH diameter $D_{\mathrm{SH}}$. The fluxes through other SHs, on the contrary, increase as $D_{\mathrm{SH}}$ decreases, but the magnitudes are always much smaller than those of SH1 and SH8. Note that the magnitude of fluxes through SH4 and SH5 in the middle of the stent falls within range of the numerical uncertainty, so were SH3 and SH6 at larger $D_{\mathrm{SH}}$ and therefore should not be interpreted as active SHs.

### 3.3 Wall shear stress

**FIGURE 4:**
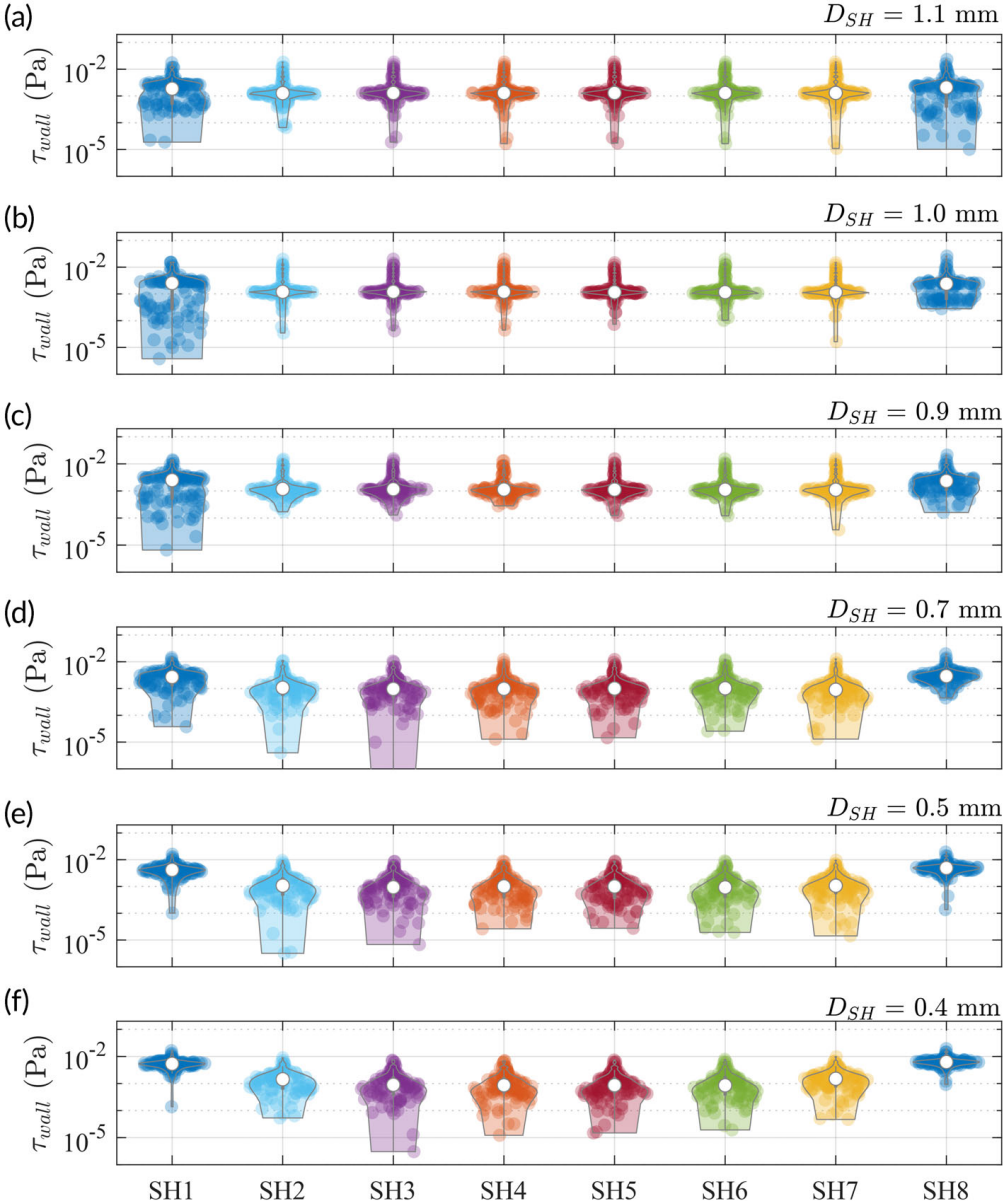
Wall shear stresses $\tau_{\text{wall}}$ at the SHs with $D_{\mathrm{SH}}=1.1,1,0.9,0.7,0.5,0.4\;\mathrm{mm}$, respectively. The violin plots show the distribution of the wall shear stress values, where the empty circles mark the median of each data set.

We continue by presenting the wall shear stress at the SHs with different $D_{\mathrm{SH}}$ using the CFD data (Figure 4). The shear stresses at the SH walls ($r\in \left[-0.5,\;0\right]\;\mathrm{mm}$) are defined as

(2) $$\begin{array}{c} \tau_{\text{wall}}=\mu {\left|\frac{\partial V}{\partial x}\right|}_{x\;=\;x_{SH}\pm \;\frac{D_{\mathrm{SH}}}{2}} \end{array}$$

where the derivatives are calculated from the velocity field using the one‐sided second‐order finite difference scheme. The violin plots in Figure 4 give the distribution of $\tau_{\text{wall}}$ and their median (empty circles) for each case. As $D_{\mathrm{SH}}$ decreases, the distribution of $\tau_{\text{wall}}$ varies such that their values at the two ends of the stent (SH1 and SH8) become more skewed at higher values, whereas their values at the middle of the stent (e.g., SH4 and SH5) spreads toward lower values.

Using the nonparametric Wilcoxon rank‐sum test, the values of $\tau_{\text{wall}}$ at SH1 are significantly smaller (p<0.001) for $D_{\mathrm{SH}}=1.1\mathrm{mm}$ (median: $1.87\times 10^{-3}\;\mathrm{Pa}$, interquartile range: $4.91\times 10^{-4}$ to $2.8\times 10^{-3}\;\mathrm{Pa}$) than for $D_{\mathrm{SH}}=0.4\;\mathrm{mm}$ (median: $5.36\times 10^{-3}\;\mathrm{Pa}$, interquartile range: $4.57\times 10^{-3}$ to $6.23\times 10^{-3}\;\mathrm{Pa}$). Meanwhile, the distribution of $\tau_{\text{wall}}$ at SH4 is higher (p<0.001) for $D_{\mathrm{SH}}=1.1\;\mathrm{mm}$ (median: $1.29\times 10^{-3}\;\mathrm{Pa}$, interquartile range: $1.10\times 10^{-3}$ to $1.79\times 10^{-3}\;\mathrm{Pa}$) than for $D_{\mathrm{SH}}=0.4\;\mathrm{mm}$ (median: $8.64\times 10^{-4}\;\mathrm{Pa}$, interquartile range: $4.49\times 10^{-4}$ to $1.68\times 10^{-3}\;\mathrm{Pa}$), although their values are both smaller compared to those at SH1 and SH8.

**FIGURE 5:**
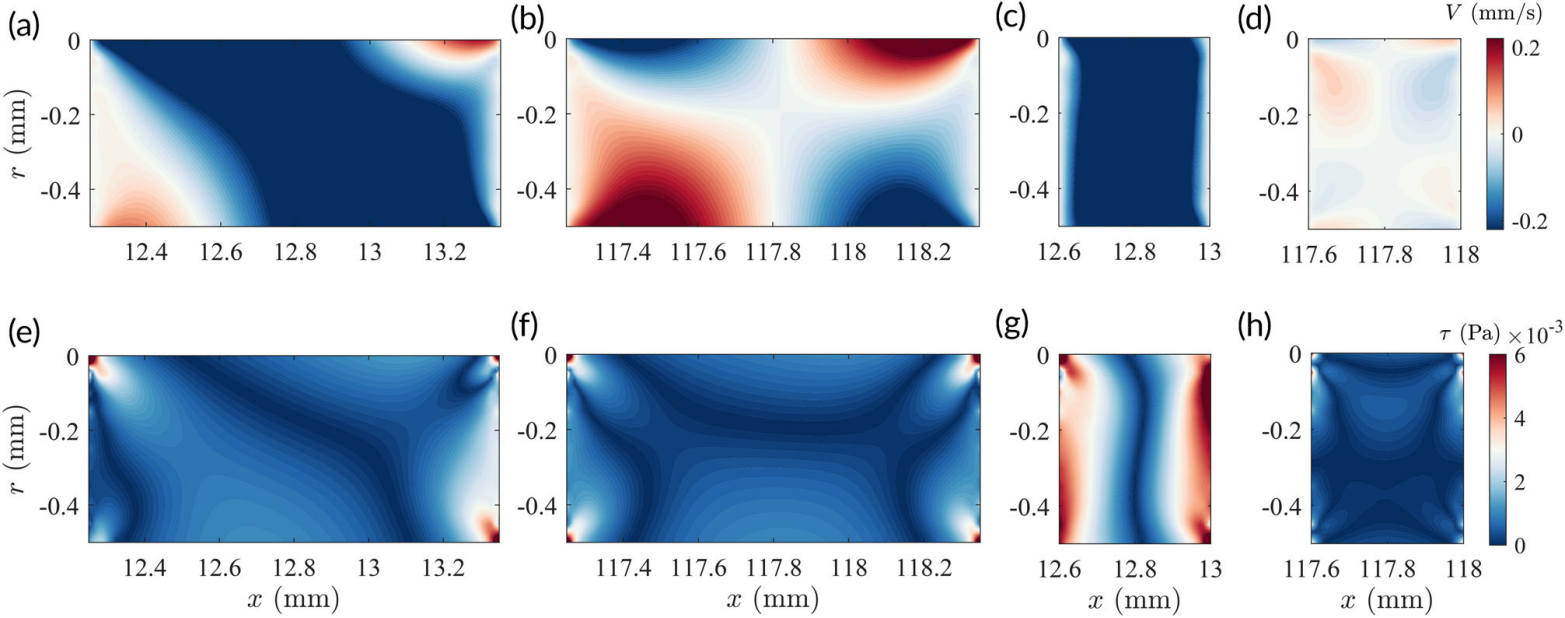
Transverse velocity fields (top row) and the corresponding shear stresses (bottom row) $\tau =\mu \left(\partial V/\partial x\right)$ in the openings for SH1 (a, e) and SH4 (b, f) from the $\;D_{\mathrm{SH}}=1.1\;\mathrm{mm}$ case, and SH1 (c, g) and SH4 (d, f) from the $D_{\mathrm{SH}}=0.4\;\mathrm{mm}$ case.

A closer examination on the flow patterns through the SHs reveals that the flux at SH1 induced by the smaller $D_{\mathrm{SH}}\;$ appears to be more uniform and parallel to the walls (Figure 5c), producing a region of high shear stresses covering the entire wall of the SH (Figure 5g). For larger $D_{\mathrm{SH}}$, the diagonal pattern of the flux produces regions of smaller wall shear stress along the SH wall (Figure 5e). At SH4, there are no appreciable fluxes (Figure 5b,d) regardless of $D_{\mathrm{SH}}$. Both the luminal and extraluminal fluids move toward the other side of the SH yet impede each other, leaving the antisymmetric flow patterns in the SH. As a consequence, regions of higher stresses only appear near the corners (Figure 5f,h).

### 3.4 Stent lumen size

**FIGURE 6:**
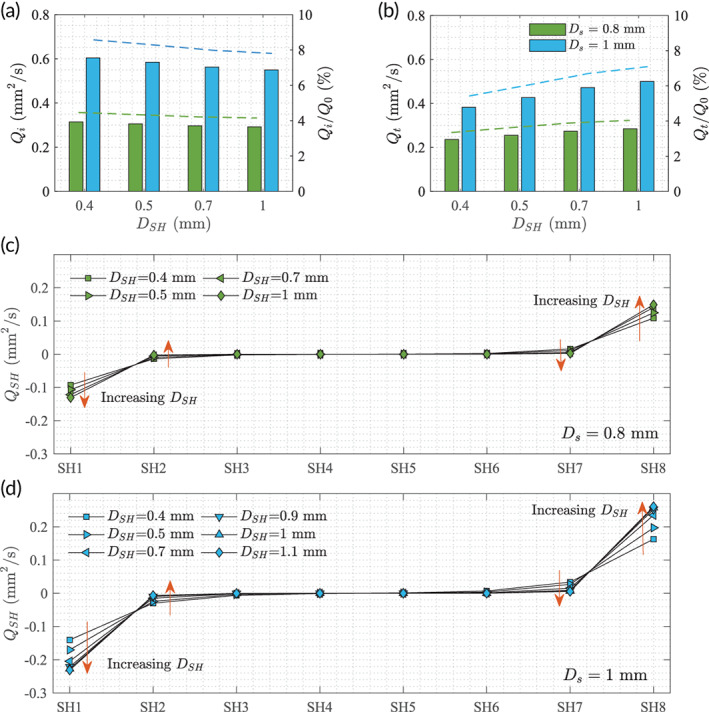
(a) Fluxes at the inlet of the stent ($Q_{i}$) and (b) the magnitude of all transverse fluxes ($Q_{t}$) with different stent lumen sizes $D_{s}$ and SH diameters $D_{\mathrm{SH}}$. The left axis gives the absolute values (bar), and the right axis gives the corresponding percentage (dashed line) normalized by the total flux $Q_{0}$. Individual fluxes ($Q_{\mathrm{SH}}$) at each SH in various cases are given in (c) and (d) for $D_{s}=0.8\;\mathrm{mm}$ and 1mm, respectively.

We complemented our study by changing the stent lumen size $D_{s}$ to evaluate the luminal flux.

(3) $$\begin{array}{c} Q_{i}=\int_{-1-\frac{D_{s}}{2}}^{-1+\frac{D_{s}}{2}}U\left(r\right)\mathrm{dr}\; \end{array}$$

at the inlet of the stent, and the magnitude of all transverse fluxes

(4) $$\begin{array}{c} Q_{t}=\sum_{n=1}^{8}\left|Q_{{\mathrm{SH}}_{n}}\right|\; \end{array}$$

were also probed to represent the stent's overall capacity to exchange fluids between the luminal and extraluminal spaces. The results show that the smaller $D_{s}$ impedes the luminal flow significantly (Figure 6a) and produces less exchange of luminal and extraluminal fluids (Figure 6b). For the same $D_{s}$, smaller $D_{\mathrm{SH}}$ facilitates more $Q_{i}$ and less $Q_{t}$. The total flux of the system $Q_{0}$ in our simulation is $7.05\;mm^{2}/s$, and the $Q_{i}$ accounts only for approximately 4% and 8% in the $D_{s}=0.8\;\mathrm{mm}$ and $D_{s}=1\;\mathrm{mm}$ cases, respectively.

The transverse fluxes at each individual SHs ($Q_{\mathrm{SH}}$) are presented in Figure 6c,d without normalization. The SHs in the middle of the stent are practically inactive regardless of the stent lumen size $D_{s}$ and the SH diameter $D_{\mathrm{SH}}$. At the two ends of the stent, fluxes increase with increasing $D_{\mathrm{SH}}$ (marked by orange arrow), and the adjacent SHs (SH2 and SH7) behave inversely.

**FIGURE 7:**
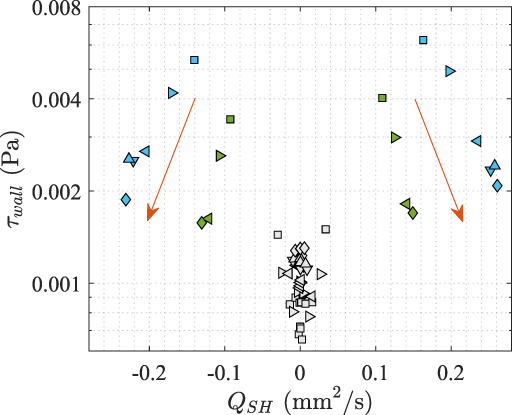
Transverse fluxes through the SHs ($Q_{\mathrm{SH}}$) versus the median wall shear stress levels at the SHs ($\tau_{\text{wall}}$) with different stent geometries. The orange arrows mark the direction of increasing $D_{\mathrm{SH}}$. Legends are the same as Figure 6, except that SH2‐7 are filled in gray to distinguish from SH1 (on the left, negative fluxes) and SH8 (on the right, positive fluxes).

Finally, we show the fluxes against the median wall shear stresses from all stent geometries tested in this study (Figure 7). The inversely related $Q_{\mathrm{SH}}$ and $\tau_{\text{wall}}$ in SHs at the two ends of the stents become clearly visible. For the same stent lumen size $D_{s}$ (blue or green), the median wall shear stress levels of the SHs increased by over 150% when the SH diameter decreased from 1.1mm to 0.4mm, whereas the flux through them decrease by about 40%. Other SHs in the middle of the stent are always associated low fluxes and low shear stresses (gray symbols in Figure 7).

## 4 DISCUSSION

Previously, 3D simulations were often used to study urinary flows with stented ureters to evaluate the impact of design parameters such as the number of SHs^11^, ^13^ and their angular position^11^ in various ureter shapes^19^ with different levels of ureteral obstruction.^14^, ^19^ Conclusions from these studies showed that (i) the total flow rate (sum of luminal and extraluminal flow rates) increases with the number of SHs,^11^ and with smaller stent size $D_{o}$,^12^ whereas angular arrangement of the SHs does not affect the total flow rate^11^; (ii) in the case of no local obstruction most of the SHs are inactive, except the ones close to the UPJ and UVJ,^11^, ^13^ and local obstruction activates the SHs directly upstream and downstream to the site.^14^, ^19^

To continue the discussion, we first show that these large‐scale flow characteristics can be derived from 0D models and scaling relations using simple fluid mechanical principals. If we approximate a stented ureter model as a straight coaxial annulus (ignoring the luminal space of stent for a moment), the bulk flow rate is given analytically by

(5) $$\begin{array}{c} Q=\frac{\pi }{128\mu }\left(-\frac{\mathrm{dp}}{\mathrm{dx}}\right)\left[D^{4}-D_{o}^{4}-\frac{{\left(D^{2}-D_{o}^{2}\right)}^{2}}{\text{In}\left(\frac{D}{D_{o}}\right)}\right] \end{array}$$

where D is the diameter of the ureter, and $D_{o}$ is the outer diameter of the stent. The pressure gradient term −∂p/∂x can be approximated by $\left(P_{0}-P_{L}\right)/L$, where $P_{0}\;\left(>0\right)$ is the relative pressure at the inlet of the ureter, $P_{L}$ is the pressure loss, and L is the ureter length. The pressure loss due to viscosity and changes of cross‐sectional shape (such as contraction or expansion of the ureter) can be approximated by^23^

(6) $$\begin{array}{c} P_{L}=P_{\text{viscosity}}+P_{\text{minor}}=f\frac{\mathrm{\rho L}}{2}\frac{{\bar{U}}^{2}}{D_{h}}+\sum_{j=1}^{M}\left(K_{j}\frac{\rho {\bar{U}}^{2}}{2}\right) \end{array}$$

where f is the friction coefficient of the surface, L is the pipe length, K is the loss coefficient, and M is the number of minor losses present in the system. In the laminar flow regime, the friction coefficient can be derived as

(7) $$\begin{array}{c} f=\frac{64\zeta }{Re_{D_{h}}} \end{array}$$

where $Re_{D_{h}}$ is the Reynolds number based on the hydraulic diameter $D_{h}=D-D_{o}$, and

(8) $$\begin{array}{c} \zeta =\frac{{\left(D-D_{o}\right)}^{2}\left(D^{2}-D_{o}^{2}\right)}{D^{4}-D_{o}^{4}-{\left(D^{2}-D_{o}^{2}\right)}^{2}/\text{In}\left(D/D_{o}\right)} \end{array}$$

**FIGURE 8:**
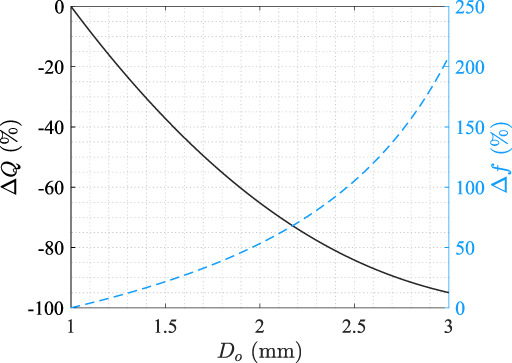
Variation of Q (solid, black) and f (dashed, blue) at various stent size $D_{o}$ relative to the case of $D_{o}=1\;\mathrm{mm}$.

Assuming D=4mm with constant ∂p/∂x and $\bar{U}$ in Equations (5) and (7), we show that by increasing the outer diameter of the stent $D_{o}$ from 1 to 3mm, the viscous loss coefficient increases by 200%, and the flow rate decreases by over 90% (Figure 8), quantifying the previous conclusion that larger stent size causes smaller total flow rate.^12^

The effect of ureter shape, such as those described as tapered or undulated in the literature,^19^ can be evaluated by the term $P_{\text{minor}}$ in Equation (6), where any change in cross‐sectional shape of the ureter, either contraction or expansion, will increase the minor loss and result in a smaller Q. This is consistent with the previous conclusion that straight ureter gives the highest total flow rate compared to the tapered and undulated shapes when other parameters are kept the same.^19^

If we consider SHs in Equation (6), more SHs are equivalent to smaller length of the wall L due to the reduced surface area, and consequently smaller $P_{\text{viscosity}}$. Meanwhile, the impact of SHs on $P_{\text{minor}}$ might be negligible since most of the SHs are inactive (Figure 6c,d). The angular rotation of the SHs does not change the pressure loss as the equivalent L and number of minor losses M are both independent of the rotation angle. That is to say, the more SHs the smaller the $P_{L}$, the larger the total flow rate Q, and that the angular rotation does not affect the total flow rate. This is again consistent with the previous conclusions from previous conclusions.^11^, ^19^

For analysis on the luminal flow rate within the stent, the inlet and outlet of the stent lumen are of primary interest. The loss coefficient K for sudden contraction ($K_{c}$ at stent inlet) and sudden expansion ($K_{e}$ at stent outlet) is given by

(9) $$\begin{array}{c} K_{c}\approx c\left(1-\frac{D_{s}^{2}}{D^{2}}\right),K_{e}={\left(1-\frac{D_{s}^{2}}{D^{2}}\right)}^{2} \end{array}$$

where $D_{s}$ is the lumen diameter of the stent ($D_{s}<D$), and c is an empirical constant.^23^ It can be inferred that the larger the $D_{s}$, the smaller the $K_{c}$ and $K_{e}$. Consequently, larger $D_{s}$ leads to less pressure loss (Equation 6) and encourages higher flow rates in the stent lumen, which explains the larger $Q_{i}$ for $D_{s}=1\;\mathrm{mm}$ (Figure 6a). The pressure loss at the stent inlet leads to the pressure imbalance between the luminal and the extraluminal spaces, which ultimately drives the activation of the SHs.

In this study, we showed that only SH1 and SH8 were always active. Since they were close to the inlet and outlet of the stent, the interluminal pressure difference re‐balances through these SHs, leaving the majority of the SHs inactive in the middle of the stent, characterized by low shear stress and low flux (Figure 7). This conclusion agrees with previous findings using 3D numerical simulations,^11^, ^13^ and perhaps explains why SHs are often heavily encrusted in clinical observations.^6^, ^7^, ^8^

The fact that only SHs close to the UPJ and UVJ are active is determined by the pressure loss at the stent inlet/outlet. The rest of the SHs can only be activated where local obstruction is present. In the ideal scenario, stents should perhaps differentiate for patients with proximal/distal obstructions, as categorized in the clinic, so that more SHs are placed near the obstruction site to improve the drainage.

If we extend the stent geometry in this study to include longer pigtails, the loss coefficient at the inlet can be scaled as $K∼s/D_{s}$, where s is the length of the protrusion part of the stent.^23^ Therefore, longer pigtails ($s/D_{s}\gg 1$) create higher pressure loss at the inlet that further impede the luminal flow and cause larger pressure difference between the luminal and the extraluminal spaces.

In fact, we show an auxiliary case study in the Supporting Information S1, where we added a pigtail to the stent model with $D_{\mathrm{SH}}=1\;\mathrm{mm}$ and $D_{s}=1\;\mathrm{mm}$. The resulting $Q_{i}$ showed a reduction by 60%, and the flux through SH1 increased by more than 100% in response to the escalated interluminal pressure difference. The smaller $Q_{i}$ in stent lumen is likely to cause local flow stasis between the inlet/outlet and the first SH in the vicinity, which promotes micro‐particle aggregations and bacterial attachments.^9^, ^10^ This perhaps explains the heavy encrustations on stent pigtails observed in several clinical studies.^6^, ^24^, ^25^, ^26^

Recent microfluidic studies^9^, ^10^ demonstrated that, by optimizing the shape of SHs to increase the local wall shear stress levels, both micro‐particle aggregation and bacterial attachment were reduced. Nonetheless, the actual shear stress level around SH largely depends on the regional flow pattern (e.g., Figure 5) that varies with the SH diameter and its longitudinal location. In Figure 7, we showed that for the same stent lumen size $D_{s}$ (blue or green) the larger the SH diameter $D_{\mathrm{SH}},$ the larger the flux $Q_{\mathrm{SH}}$, and the smaller the median wall shear stress $\tau_{\text{wall}}$, which was explained by the flow pattern within the SH (Figure 5). It demonstrated the importance of full scale simulations with the focus to resolve detailed flow characteristics to understand the interactions between scales.

In terms of lumen diameter of the stent, larger $D_{s}$ promoted both luminal flow rate and the interluminal exchange of fluid (Figure 6). The latter was mainly contributed by the active SHs. Based on Equation (9), the larger lumen diameter helped alleviate the pressure loss at the inlet/outlet of the stent and thus encouraged more luminal flux. Nonetheless, the luminal flux only accounted for up to 9% of the total flux in our study and further reduced down to ~3% when the pigtail was included (see Supporting Information S1). In this regard, further studies should focus on evaluating stent pigtails with different inlet designs and lumen diameters, balancing the antidislocation function and its impact on the stent performance for different types of patient.

In summary, our results suggest that larger SH and larger lumen size should be chosen where internal obstruction is the primary concern since they promote better drainage capability and encourage interluminal exchange of fluids. For long‐term stenting (e.g., during pregnancy) where encrustation is the primary concern, smaller SH seems to be a better choice since more SHs are activated and shear stress levels on the SHs close to the UPJ and UVJ are higher. Smaller SH also means better tensile strength against radial compression so seems to suit patients with extrinsic obstructions. Further comparative evaluations with other stent designs such as those of noncircular cross sections^27^, ^28^ might be of interest.

To close the discussion, we acknowledge the major limitations of the current study. First, even though the role of pigtail was inferred in the discussion and briefly visited in the Supporting Information S1, full documentation on its impact on the flow characteristics especially with different design parameters is still desirable. Second, the ureter was modeled as a straight rigid tube in this study with constant cross‐sectional geometry, where the elasticity and tapering of real ureters are not modeled. These factors can be evaluated using previous results on reduced order models of the urinary tract.^18^ Further, the vesicoureteral reflux, which is the flux of urine from bladder to the ureter(s) during bladder contraction, was not investigated in this study. Such retrograde flow will impose dynamic changes to the flow characteristics near the UVJ. Although, the relevant changes caused by this pressure rise should be alleviated by including more SHs in the distal part of the stent according to our discussions. The stented ureter poses a complex multidisciplinary system, and patient‐specific physiochemical environment is an important factor to address. Nevertheless, the results from current study offered a glimpse into the baseline fluid mechanical principles of the system and could be used to guide further studies of the system with more complex physiochemical conditions.

In short, the “perfect stent” should be more patient oriented, not necessarily individualized but should be designed to address the primary need in each type of patient.

## AUTHOR CONTRIBUTIONS

**Shaokai Zheng:** Conceptualization (equal); data curation (equal); formal analysis (equal); investigation (equal); methodology (equal); resources (equal); software (equal); validation (equal); visualization (equal); writing – original draft (equal). **Dominik Obrist:** Conceptualization (equal); funding acquisition (equal); project administration (equal); resources (equal); supervision (equal); writing – review and editing (equal). **Fiona Burkhard:** Conceptualization (equal); funding acquisition (equal); project administration (equal); resources (equal); supervision (equal); writing – review and editing (equal). **Francesco Clavica:** Conceptualization (equal); funding acquisition (equal); project administration (equal); resources (equal); supervision (equal); writing – review and editing (equal).

## CONFLICT OF INTEREST

The authors declare that the research was conducted in the absence of any commercial or financial relationships that could be construed as a potential conflict of interest.

### PEER REVIEW

The peer review history for this article is available at https://publons.com/publon/10.1002/btm2.10407.

## Supporting information


**Appendix S1** Supporting Information

## Data Availability

The data that support the findings of this study are available on request from the corresponding author. The calibration code used in the experiment can be found on Github at https://github.com/zheng-sk/. Further information and supplementary data can be found in Supporting Information.
