# Supplementary material for: Fluid mechanical performance of ureteral stents: The role of side hole and lumen size
Source: Bioeng Transl Med. 2022 Sep 13;8(2):e10407. doi: 10.1002/btm2.10407 (PMC10013766; doi:10.1002/btm2.10407)
Supplement: Supplementary file 1 — Appendix S1 Supporting Information [file BTM2-8-e10407-s001.pdf]

SUPPORTING INFORMATION

Fluid mechanical performance of ureteral stents: The role of side hole and lumen size

Shaokai Zheng[1,§], Dominik Obrist[1], Fiona Burkhard[2], and Francesco Clavica[1,2]

[1]ARTORG Center for Biomedical Engineering Research, Faculty of Medicine, University of Bern, Bern, Switzerland
[2] Department of Urology, Inselspital, Bern University Hospital, University of Bern, Bern, Switzerland

1 Experimental methods

1.1 Calibration

The imaging system was calibrated using a planar calibration plate with circular dot patterns of 0.125 mm diameter and 0.25 mm spacing (R2L2S3P2, Thorlabs Inc., NJ, US). Since the calibration plate cannot fit in the ureter model, an ex-situ calibration procedure was developed, similar to that in (1). First, a half replica of the ureter model, referred to as the half pipe, was made following the same manufacturing procedure as for the ureter model. The calibration plate was mounted in the acrylic box, aligning with the median plane ($x - r$ plane at $z = 0$ mm), and the half pipe was placed in front of the calibration plate to simulate the optical path through the ureter model (figure S1A). The box was then filled with the working fluid, and 10 images were acquired.

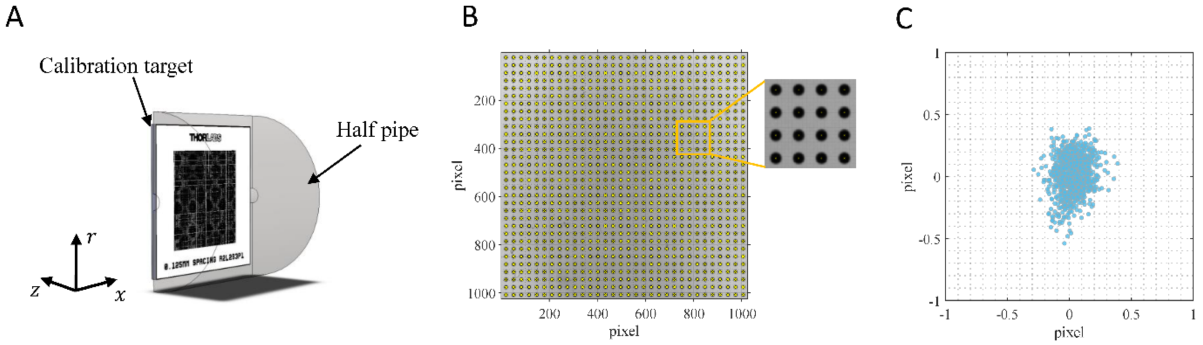

Figure S1. (A) Illustration of the ex-situ calibration method, where a half-replica of the ureter model was placed in front of the calibration plate to simulate the actual optical path. (B) Example of a calibration image, where center of the circular targets are extracted and marked by the yellow cross. (C) Re-projection error of the image in (B), and the RMS error is 0.17 px.

§ Correspondence: Shaokai Zheng, ARTORG Center for Biomedical Engineering Research, Faculty of Medicine, University of Bern, Freiburgstrasse 3, 3010 Bern, Switzerland. Email address: shaokai.zheng@outlook.com

To calculate the camera matrix, a code was developed in-house using the Computer Vision Toolbox™ from MATLAB (R2020b, MathWorks, MA, US), which takes one view of the calibration plate and utilizes an iterative optimization procedure to minimize the re-projection error. The process is illustrated in figure S2. The image coordinates $(\mathbf{x},\ \mathbf{y})$ of the center of the circular pattern, referred to as the image centers, were extracted (figure S1B) using the Circular Hough transform (2), and the world coordinates $(\mathbf{X},\ \mathbf{Y})$ were generated from the specification of the calibration target, referred to as the world centers. The two sets of coordinates were used to compute the Homography. Assuming a fixed principal point $(c_x,\ c_y)$ and zero skew, the effective focal length of the system $(f_x,\ f_y)$ was calculated along with the rotation and translation matrix $[\mathbf{R}, \mathbf{t}]$. The camera matrix was then constructed as

$$M = \begin{bmatrix} f_x & 0 & c_x \\ 0 & f_y & c_y \\ 0 & 0 & 1 \end{bmatrix} [\mathbf{R} \quad \mathbf{t}]. \tag{S1}$$

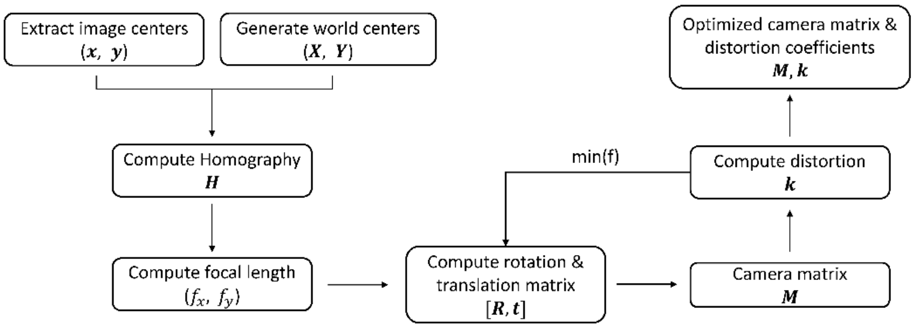

Figure S2. Flow chart of the calibration process.

Following that, the ideal image centers using the estimated camera matrix can be calculated by

$$\tilde{\mathbf{x}} = \mathbf{MX}. \tag{S2}$$

The distorted image centers $(\hat{x},\ \hat{y})$ was given by

$$\begin{aligned} \hat{x} &= \tilde{x} + (\tilde{x} - c_x)[(k_1(\tilde{x}^2 + \tilde{y}^2) + k_2(\tilde{x}^2 + \tilde{y}^2)^2] \\ \hat{y} &= \tilde{y} + (\tilde{y} - c_y)[(k_1(\tilde{x}^2 + \tilde{y}^2) + k_2(\tilde{x}^2 + \tilde{y}^2)^2]' \end{aligned} \tag{S3}$$

where k is the distortion coefficient. The tangential distortion was neglected since the distortion function is dominate by the radial components (3). Equation S3 can be rearranged into the matrix form $\mathbf{Ak} = \mathbf{B}$ such that

$$\begin{bmatrix} (\tilde{x} - c_x)(\tilde{x}^2 + \tilde{y}^2) & (\tilde{x} - c_x)(\tilde{x}^2 + \tilde{y}^2)^2 \\ (\tilde{y} - c_y)(\tilde{x}^2 + \tilde{y}^2) & (\tilde{y} - c_y)(\tilde{x}^2 + \tilde{y}^2)^2 \end{bmatrix} \begin{bmatrix} k_1 \\ k_2 \end{bmatrix} = \begin{bmatrix} \hat{x} - \tilde{x} \\ \hat{y} - \tilde{y} \end{bmatrix} \tag{S4}$$

where $\mathbf{k} = [k_1,\ k_2]^T$. The solution is given by

$$\mathbf{k} = \left(\mathbf{A}^T\mathbf{A}\right)^{-1}\mathbf{A}^T\mathbf{B}. \tag{S5}$$

Once the camera matrix and distortion coefficients were both estimated, the world centers was re-projected into the image coordinates, and the error was minimized by

$$\min_{\mathbf{M},\mathbf{R},\mathbf{t},k_1,k_2} \sum_{p=1}^{n}\sum_{q=1}^{m}\sqrt{\left(\hat{x}_{p,q}-x_{p,q}\right)^2+\left(\hat{y}_{p,q}-y_{p,q}\right)^2} \quad (S6)$$

where p, and q are the column and row index, respectively, and n and m are the number of column and rows of control points in the image. The final RMS error (e.g. figure S1C) of the calibration was evaluated by

$$\epsilon = \sqrt{\frac{1}{P}\sum_{p,q=1}^{P}\left(\left(\hat{x}_{p,q}-x_{p,q}\right)^2+\left(\hat{y}_{p,q}-y_{p,q}\right)^2\right)}. \quad (S7)$$

In this study, the RMS error was ~ 0.2 px on average (range $0.1-0.3$ px), which incorporated both the calibration error and the residual error caused by the refractive index matching. The limiting factor in this study was the refractive index matching, which produced errors between $0.1-0.2$ px. Nevertheless, the calibration procedure produce results better than the linear scaling method implemented in most open source PIV processing packages. The code will be made available on Github at https://github.com/zheng-sk/, and might be used in occasions where multi-view calibration is not possible due to confined spaces.

1.2 PIV processing

After acquisition, the intensity level of individual images was normalized to the entire range of the bit-depth (8-bit), and highpass-filtered at 70% of the highest intensity to remove dark particles. A background removal method was applied afterwards to eliminate the particles settled on the walls as reported by (4).

Due to the relatively small Field of View (FOV) to the total volume of the fluid, the number of particles in the FOV was low to keep the cost per experiment at a reasonable level. Therefore, an image overlapping method was implemented as a pre-processing step. This was done under the assumption of a steady flow, and might be extended to include phased-locked measurement of pulsatile flows.

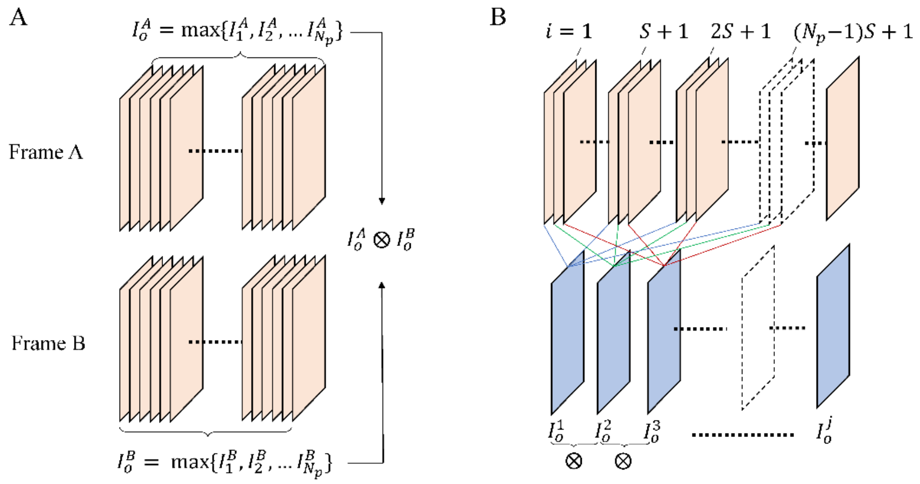

Figure S3. (A) Illustration of previously proposed image overlapping method (5). (B) Illustration of the image overlapping method proposed in this study, where N_p is the number of overlapped image, S is the number of skipped image, i is the index of original images, and j is the index of overlapped image.

In previous literature, a similar strategy was proposed by overlapping uncorrelated image pairs with a max filter to increase the particle image density as illustrated in figure S3A (5). For an image pair I_i^A and I_i^B, where $i = 1, 2, \dots N$ is the image index, the max filter was applied as $I_o^A = \max\left\{I_1^A, I_2^A, \dots I_{N_p}^A\right\}$ and $I_o^B = \max\left\{I_1^B, I_2^B, \dots I_{N_p}^B\right\}$, where $\mathrm{N_p}$ is the number of images to overlap, and $\mathrm{I_o^A}$ and $\mathrm{I_o^B}$ are the resulting images for subsequent cross-correlation. For continuous image series acquired by high speed imaging systems, however, the method leads to streaks of particles as demonstrated in figure S4A and B. We addressed this by adopting a skipping-overlapping method such that for an image series I_i, where $i = 1, 2, \dots N$, the new overlapped series of images are produced as $I_j = \max\left\{I_1, I_{S+1}, I_{2S+1}, \dots I_{(N-1)S+1}\right\}$, where S is the number of skipped image, and j is the index of resulting images. The current method reduces to the previous method when $S = 1$.

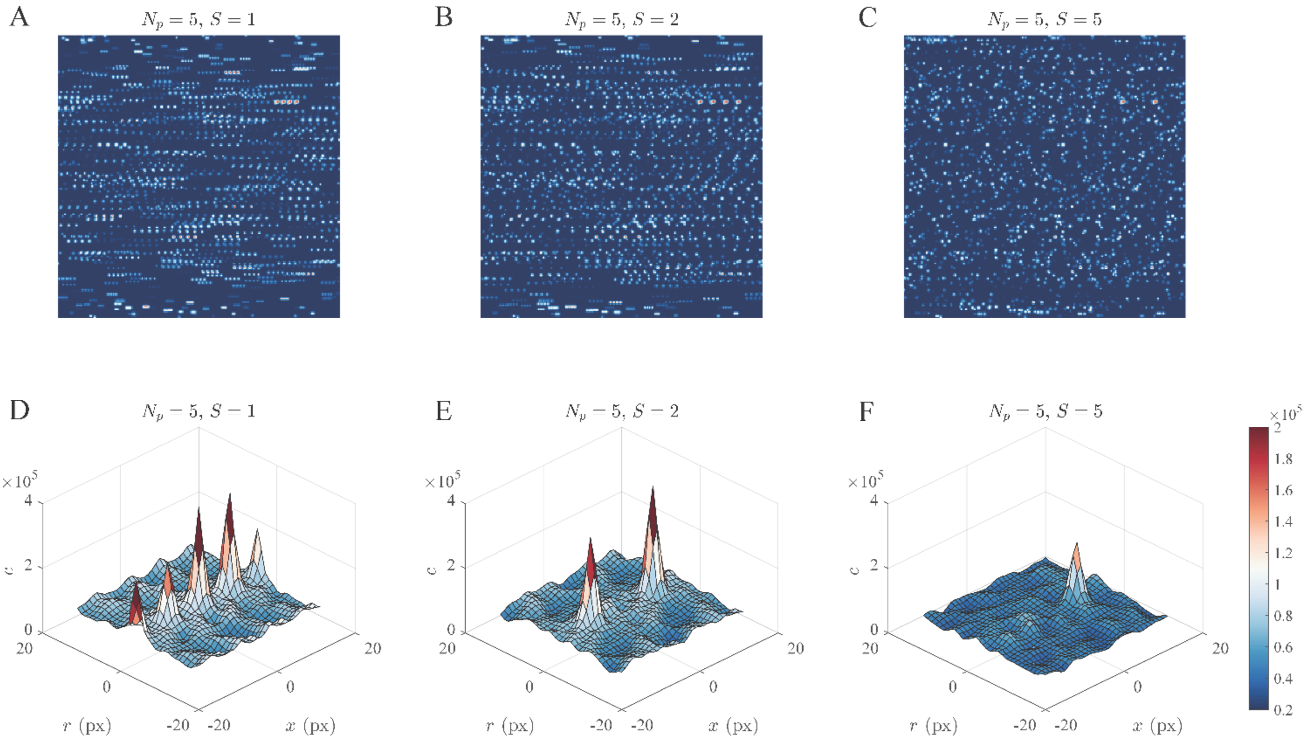

Figure S4. Examples of the image overlapping method with increasing S (A-C). Correlation maps at the center ($32 \times 32\ px$) of the images are given in (D-F).

Examples of the overlapped images are given in figure S4A-C, where synthetic image series (256×256 px) of a Poiseuille flow are overlapped with different values of S. Figure S4A ($S = 1$) is equivalent to the previous method, and in this case introduces streaks of particle images, which leads to multiple false correlation peaks along the flow direction (figure S4D). The issue was remedied by increasing the value of S (figure S4C), which gives only one definite correlation peak with less noise (figure S4F). In practice, the value of S can be estimated by the ratio between the interrogation window size and the smallest velocity in a given flow field, such that the same particle does not appear in the interrogation window twice. Discussions on the choice of $\mathrm{N_p}$ can be found in the work of (5, 6).

After overlapping, the resulting images were processed using the PIVlab (7). Velocity fields were calculated using the ensemble-correlation with interrogation window of 64×64 px for the first pass and 32×32 px for the second, both with 50% overlap. The sub-pixel resolution was calculated using

the three-point Gaussian interpolation. The velocity field was filtered by the outlier detection algorithm from (8), and filled by the moving median of the 3×3 neighboring points.

2 Stent with pigtail

In this section, we show the auxiliary case study by adding a pigtail to the stent model. The geometries of the original stent and the one with pigtail are shown in figure S5 along with the streamwise velocity and pressure for each case. The luminal diameter of the stent is $\mathrm{D_s} = 1\ \mathrm{mm}$ for both cases, and so was the SH diameter $\mathrm{D_{SH}} = 1\ \mathrm{mm}$. The pigtail is modeled by extending the stent inlet into a loop. From the pressure field figure S5C, D, it is obvious that the pressure in the renal pelvis is larger when the pigtail is present, and the pressure difference between the pigtail lumen and the renal pelvis is larger.

	$\mathrm{Q_i}$ (%)	$\mathrm{Q_{SH_1}}$ (%)	$\mathrm{Q_{SH_2}}$ (%)
Straight	0.55 (7.8)	0.23 (3.2)	0.0079 (0.11)
Pigtail	0.22 (3.1)	0.55 (7.8)	0.019 (0.27)

Table S1 The magnitude of flux (in $\mathrm{mm^2/s}$) through the stent inlet ($\mathrm{Q_i}$) and through the first two SHs ($\mathrm{Q_{SH_1}}$ and $\mathrm{Q_{SH_2}}$). The percentage shows the flux normalized by the total flux.

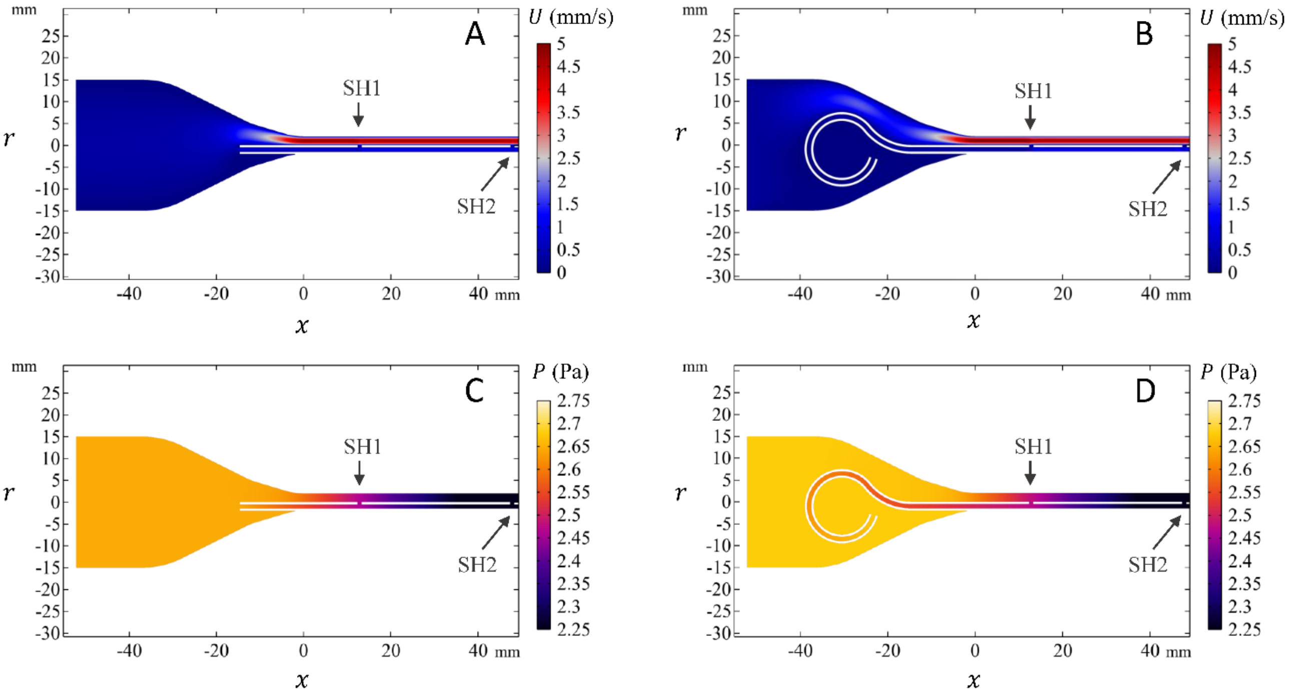

Figure S5. Comparison of streamwise mean velocity (top row) and pressure (bottom row) for the original stent geometry (A and C) as reported in the main text, and the Auxiliary case with extended pigtail (B and D).

To quantify the difference the velocity profiles across the stent lumen at $\mathrm{x} = 0\ \mathrm{mm}$ were extracted and integrated across the lumen to give the luminal flux $\mathrm{Q_i}$. Results are compared in table S1. As

discussed in the main text, the pigtail caused 60% reduction in the luminal flux, which was equivalent to 4.7% of the total flux in the ureter. Meanwhile, the flux through the first two SHs were both increased by more than 100% due to the escalated pressure difference between the luminal and extraluminal spaces.

Note that the 2D simulation inevitably exaggerates the effect of the pigtail as the velocity in z is forced to be zero, although in practice the kidney side of the stent is tapered into a nozzle to ease the insertion, which increases the pressure loss at the inlet. As discussed in the main text and demonstrated here, the fluid mechanical characteristics of the pigtail and strategies to reduce its associated pressure loss seem to be desirable for future stent developments.

REFERENCES

1. Gunasekera S, Ng O, Thomas S, Varcoe R, de Silva C, Barber T. Tomographic PIV analysis of physiological flow conditions in a patient-specific arteriovenous fistula. Experiments in Fluids. 2020;61(12):253.

2. Yuen HK, Princen J, Illingworth J, Kittler J. Comparative study of Hough Transform methods for circle finding. Image and Vision Computing. 1990;8(1):71-7.

3. Zhang Z. A flexible new technique for camera calibration. IEEE Transactions on Pattern Analysis and Machine Intelligence. 2000;22(11):1330-4.

4. Deen NG, Willems P, van Sint Annaland M, Kuipers JAM, Lammertink RGH, Kemperman AJB, et al. On image pre-processing for PIV of single- and two-phase flows over reflecting objects. Experiments in Fluids. 2010;49(2):525-30.

5. Nguyen CV, Fouras A, Carberry J. Improvement of measurement accuracy in micro PIV by image overlapping. Experiments in Fluids. 2010;49(3):701-12.

6. Westerweel J. Theoretical analysis of the measurement precision in particle image velocimetry. Experiments in Fluids. 2000;29(1):S003-S12.

7. Thielicke W, Sonntag R. Particle Image Velocimetry for MATLAB: Accuracy and enhanced algorithms in PIVlab. Journal of Open Research Software. 2021;9(12).

8. Westerweel J, Scarano F. Universal outlier detection for PIV data. Experiments in Fluids. 2005;39(6):1096-100.
